# 

**Published:** 1898-12

**Authors:** 


### BOOKS RECEIVED.

Materia Medica, Pharmacy, Pharmacology and Therapeutics. By W. Hale White, M. D., F. R. C. P., Physician to, and Lecturer on Pharmacology and Therapeutics at Guy's Hospital, London; Examiner in Materia Medica to the University of London, etc., etc. Edited by Reynold W. Wilcox, M. A., M. D., LL. D., Professor of Medicine and Therapeutics at the New York Post-Graduate Medical School, and Attending Physician to the Post-Graduate Hospital, etc., etc. Fourth American edition. Thoroughly revised. Small 8vo, pp. 704. Price, $3.00. Philadelphia: P. Blakiston's Son & Co. 1898.

Human Anatomy. A Complete, Systematic Treatise by Various Authors. Including a Special Section on Surgical and Topographical Anatomy. Edited by Henry Morris, M. A., M. B., Lond. Senior Surgeon to the Middlesex Hospital; Examiner in Surgery in the University of London; Member of the Council and Chairman of the Court of Examiners of the Royal College of Surgeons of England; Honorary Member of the Medical Society of the County of New York. Illustrated by 790 wood cuts, the greater part of which are original and made expressly for this work by special artists, over 200 of which are printed in colors. Second edition, revised and enlarged. Royal 8vo pp. xxx.—1274. Price, cloth, $6.00. Philadelphia: P. Blakiston's Son & Co. 1898.

Transactions of the Pathological Society of Philadelphia. Vol. XVIII. Containing the Report of the Proceedings of the Society for October, 1895, to June, 1897. Edited by William S. Carter, M. D., Recorder of the Society. Octavo, pp. 493. Philadelphia: William J. Dornan, Printer. 1898.

International Clinics. A Quarterly of Clinical Lectures and Specially Prepared Articles on Treatment and Drugs. By professors and lecturers in the leading medical colleges of the United States, Germany, Austria, France, Great Britain and Canada. Edited by Judson Deland, M D., (Univ. of Penna.), Philadelphia, Instructor in Clinical Medicine and Lecturer on Physical Diagnosis in the University of Pennsylvania; Assistant Physician to the Hospital of the University of Pennsylvania, etc.; Fellow of the College of Physicians of Philadelphia. J. Mitchell Bruce, M. D., F. R. C. P., London, England, Physician to and Lecturer on the Principles and Practice of Medicine at the Charing Cross Hospital. David W. Finley, M. D., F. R. C. P., Aberdeen, Scotland, Professor of Practice of Medicine in the University of Aberdeen; Physician to and Lecturer on Clinical Medicine in the Aberdeen Royal Infirmary, etc. Volume III. Eighth Series. 1898. Octavo, pp. xii.—355. Philadelphia: J. B. Lippincott Co. 1898.

A Manual of the Practice of Medicine. By Frederick Taylor, M. D., F. R. C. P. Physician to and Lecturer on Medicine at Guy's Hospital; Consulting Physician to the Evelina Hospital for Sick Children; Examiner in Medicine at the University of London, etc. etc. Fifth edition. Small 8vo, pp. xvi.—1002. Price, $4.00. London: J. & J. A. Churchill, 7 Great Marlborough St. Philadelphia: P. Blakiston's Son & Co. 1898.

A Pocket Medical Dictionary Giving the Pronunciation and Definition of the Principal Words used in Medicine and the Collateral Sciences. By George M. Gould, A. M., M. D, Author of The Illustrated Medical Dictionary, The Student's Medical Dictionary; Editor of the Philadelphia Medical Journal, etc. New edition, rewritten and enlarged, including over 21,000 words. Philadelphia: P. Blakiston's Son & Co. 1898.

A Compend of Obstetrics, Especially Adapted to the Use of Medical Students and Physicians. By Henry G. Landis, A. M., M. D. Revised and edited by William H. Wells, M. D., Adjunct Professor of Obstetrics and the Diseases of Infancy in the Philadelphia Polyclinic, etc., etc. Sixth edition, illustrated. Quiz Compends No. 5. Philadelphia: P. Blakiston's Son & Co. 1898.

Diseases of the Skin. An Outline of the Principles and Practice of Dermatology. By Malcolm Morris, F. R. C. S., Surgeon to the Skin Department, St. Mary's Hospital, London. New (2d) edition. Revised and enlarged. In one 12mo volume of 601 pages, with 10 colored plates and 26 engravings. Cloth, $3.25, net. Philadelphia and New York: Lea Brothers & Co., Publishers.

